# The impact of the COVID-19 pandemic on rabies reemergence in Latin America: The case of Arequipa, Peru

**DOI:** 10.1371/journal.pntd.0009414

**Published:** 2021-05-21

**Authors:** Brinkley Raynor, Elvis W. Díaz, Julianna Shinnick, Edith Zegarra, Ynes Monroy, Claudia Mena, Micaela De la Puente-León, Michael Z. Levy, Ricardo Castillo-Neyra

**Affiliations:** 1 Department of Biostatistics, Epidemiology & Informatics, Perelman School of Medicine at University of Pennsylvania, Philadelphia, Pennsylvania, United States of America; 2 Zoonotic Disease Research Lab, One Health Unit, School of Public Health and Administration, Universidad Peruana Cayetano Heredia, Lima, Perú; 3 Gerencia Regional de Salud de Arequipa, Ministerio de Salud, Arequipa, Perú; 4 Red de Salud Arequipa Caylloma, Ministerio de Salud, Arequipa, Perú; Centers for Disease Control and Prevention, UNITED STATES

## Abstract

In Latin America, there has been tremendous progress towards eliminating canine rabies. Major components of rabies elimination programs leading to these successes have been constant and regular surveillance for rabid dogs and uninterrupted yearly mass dog vaccination campaigns. Unfortunately, vital measures to control COVID-19 have had the negative trade-off of jeopardizing these rabies elimination and prevention activities. We aimed to assess the effect of interrupting canine rabies surveillance and mass dog vaccination campaigns on rabies trends. We built a deterministic compartment model of dog rabies dynamics to create a conceptual framework for how different disruptions may affect rabies virus transmission. We parameterized the model for conditions found in Arequipa, Peru, a city with active rabies virus transmission. We examined our results over a range of plausible values for R_0_ (1.36–2.0). Also, we prospectively evaluated surveillance data during the pandemic to detect temporal changes. Our model suggests that a decrease in canine vaccination coverage as well as decreased surveillance could lead to a sharp rise in canine rabies within months. These results were consistent over all plausible values of R_0_. Surveillance data from late 2020 and early 2021 confirms that in Arequipa, Peru, rabies cases are on an increasing trajectory. The rising rabies trends in Arequipa, if indicative to the region as whole, suggest that the achievements made in Latin America towards the elimination of dog-mediated human rabies may be in jeopardy.

## Author summary

Effective strategies to control and eliminate canine rabies (and thus dog-transmitted human rabies) are uninterrupted mass dog vaccination campaigns and surveillance for rabid dogs. However, due to the COVID-19 pandemic, these vital control activities have been diminished or suspended due to restrictions implemented to decrease the spread of SARS-CoV-2 and redirection of funds. Almost a year after Peru’s response to COVID-19 began, canine rabies cases in Arequipa, a city with ongoing rabies virus transmission, are increasing rapidly. In recent months, reported canine rabies cases have surpassed previous years’ averages despite lower than average sample submissions. A mathematical model describes the mechanisms that might have created this rising trend: COVID-19-associated disruptions of mass dog vaccination campaigns and surveillance activities. As the COVID-19 pandemic is presenting obstacles to rabies elimination programs across Latin America, it is very likely that the region will face an increased canine rabies burden in the coming months to years if control efforts continue to be delayed, diminished, or suspended.

## Introduction

During the last decades, enormous progress has been achieved towards the elimination of canine rabies in the Americas [1–3]. By 2019, health authorities in the Americas felt that Latin America was closer than ever to achieving the elimination of human deaths by dog-mediated rabies. The Pan American Health Organization (PAHO) announced on the eve of World Rabies Day– 28 September that only five human cases were reported in the region in the previous 12 months [2]. These achievements were due mainly to a coordinated regional plan that involved multi-pronged strategies and continuous activities conducted by national governments and local communities [3]. The COVID-19 pandemic caused by the SARS-CoV-2 coronavirus has disrupted these strategies and activities in Latin America and jeopardizes the elimination prospects in the whole region.

Among the different strategies to prevent human rabies around the world, the most effective is mass dog vaccination [1,4,5]. In most Latin American countries, the Ministries of Health, Ministries of Agriculture, or other public health agencies organize annual or biannual mass canine rabies vaccination campaigns in areas affected and unaffected by canine rabies [3]. Surveillance is also a vital component of rabies control programs [6–8]. In Latin America, since 1983, rabies control programs have included regular surveillance [3]. Importantly, rabies surveillance also activates control measures in response to the report of a suspected or confirmed rabid dog, which include broad control and prevention measures such as removal of the rabid dog, dog vaccinations, administration of post-exposure prophylaxis, and removal of exposed (bitten) dogs [9–12]. Risk of SARS-CoV-2 transmission and sequential efforts to minimize that risk have presented barriers for implementation of rabies control strategies.

SARS-CoV-2 has infected more than 46.2 million people in the Americas as of February 3, 2021, and the pandemic has required an unprecedented, coordinated effort among national public health ministries [13–15]. Public health ministries have necessarily shifted their focus and resources to implementing stay-at-home orders and ramping up emergency preparedness efforts. Moreover, in Peru, veterinarians and other authorities have considered dog vaccination a high-risk activity during the pandemic or a non-essential veterinary activity (e.g. non-urgent or non-emergency care) [16]. In the case of rabies in Peru, and other Latin American countries, the yearly mass dog vaccination campaigns, the cornerstone of rabies prevention, were postponed and downsized in 2020 [17–21]. Surveillance and focus control efforts have also been scaled back due to the COVID-19 pandemic. Here we use a deterministic compartment model to explore the long-term effects of short-term changes to the rabies prevention protocols that have been developed and maintained over the past three decades. Specifically, we investigate how a reduction in canine vaccination coverage, decreased rabies surveillance, and decreased focus control efforts can affect canine rabies dynamics in Arequipa, Peru. We report a considerable and sudden increase in the number of rabid dogs in Arequipa, consistent with model trends, after failure to implement yearly mass dog vaccination campaigns in an area with reemergent transmission of rabies virus.

## Methods

### Data

Data was collected from two sources. First, from 2015–2019, a door-to-door longitudinal survey was conducted in Arequipa, Peru to capture demographic data on the dog population and on vaccination campaign participation [22]. Second, collaborators at the Ministry of Health provided epidemic data on samples submitted to the Ministry of Health and subsequent focus control reports for the positive samples. Case counts and dates associated with cases were extracted from these data including: date of first clinical signs, date of death, date of sample submission, date of positive sample confirmation, and date of focus control activities.

### Model description

We created a deterministic compartment model of canine rabies virus transmission in Arequipa, Peru. The model distributes the canine population between 4 population states- vaccinated (in yearly vaccination campaigns), susceptible, exposed (via the bite of a rabid dog) and infectious (Figure A in S2 Appendix). Equations depicting the movement between compartments can be expressed as:

$$dS/dt=\theta -\beta SI/N-\mu S-\nu_{1}\;S+\nu_{2}V$$ (1)

dE/dt=βSI/N−γE−μE (2)

dI/dt=γE−μI−αI (3)

$$dV/dt=\nu_{1}\;S-\nu_{2}V-\mu V$$ (4)

Parameters were estimated based on population means obtained from dog population surveys conducted by our group and epidemic data provided by the Ministry of Health (Table 1). A full description of parameter estimation can be found in the supplementary information (Text A in S2 Appendix). Simulations were run for a range of plausible values of R_0_ (1.36–2.0). The model was fit with data collected prior to COVID-19 restrictions (Figure B in S2 Appendix), then simulations were run for 1 year from March 2020 to March 2021 and for 5 years from March 2020 to March 2025. The 1-year model simulation results were then compared with recent prospective surveillance data from that period (March 2020- March 2021) from the Ministry of Health. All computation was done using R [23].

**Table 1:** Model Parameters.

Parameter	Definition	Estimate	Source
N	Total dog population (S+E+I+V)	203183	Arequipa Ministry of Health [24]
θ	Instantaneous per capita birth rate	θ = μN + α	Calculated to maintain a steady state equilibrium
μ	Instantaneous per capita death rate (not attributable to rabies)	1/1099.20	Calculated from survey data
γ	Instantaneous per capita rate of exposed dogs becoming infectious	1/22.3	Hampson, 2009 [25]
α	Instantaneous per capita death rate of rabid dogs attributable to rabies	1/2.53	Calculated from focus control data
ν_*1*_	Per capita vaccination rates	Changes based on yearly vaccination coverage (Table A in S2 Appendix)	Calculated from survey data
ν_*2*_	Instantaneous per capita loss of immunity rates	1/365	Nobivac and Peru Centro Nacional de Productos Biológicos [26,27]
β	Transmission coefficient	R_0_(γ+μ) (μ +α)/γ	Calculated based on next generation matrix methods [28]
R_0_	Basic reproductive number	1.44 (a range from 1.36 to 2 is presented)	Fit epidemic data

### Disruption of the rabies control program

The COVID-19 pandemic interrupted two key rabies elimination activities: mass dog vaccination and canine rabies surveillance. The disruption of each of these activities affects several parameters in the model. Many vaccination programs around the world have been affected by scarce funds already shifted towards pandemic response and fear of being infected with the COVID-19 virus [29]. Similarly, for canine rabies in Latin America, the yearly vaccination campaigns were skipped, delayed, and diminished in multiple countries in 2020 [17–21]. To examine the effects of vaccination interruption in the model we changed *v*_*1*_, the instantaneous per capita vaccination rate to reflect different scenarios: meeting the regional [30] and national goal of 80% coverage [11], a complete cancellation scenario of 0% coverage, and an intermediate effort of 58% coverage to match rates obtained previously (Table A in S2 Appendix) [22].

Changes in city life during quarantine may also have impacted rabid dogs’ survival in several ways. First, fewer people left home; in Arequipa, many rabid dogs (25%) are reported by unrelated city dwellers as opposed to dog owners [31]. Fewer people out in public compounded with increased difficulty in travel to and from health posts and disrupted health post hours led to reports of suspected rabid dogs decreasing to almost zero. Second, even if rabid dogs were reported, COVID-19 protocols disrupted rabies response teams, delaying euthanization and removal of suspected rabid dogs. Third, a decrease in vehicle traffic during the COVID-19 lockdown led to increased survival time of disoriented dogs that otherwise would have been hit by cars. Hampson et al. found that rabid dogs died of the disease in an average of 3.7 days if they were not killed [25]. Therefore, to examine the effect of increased survival time of rabid dogs due to COVID we shifted the death rate (α) from reflecting a mean survival time of 2.5 days (calculated from focus control reports) to 3.7 days (the expected survival time without focus control).

## Results

**Fig 1:**
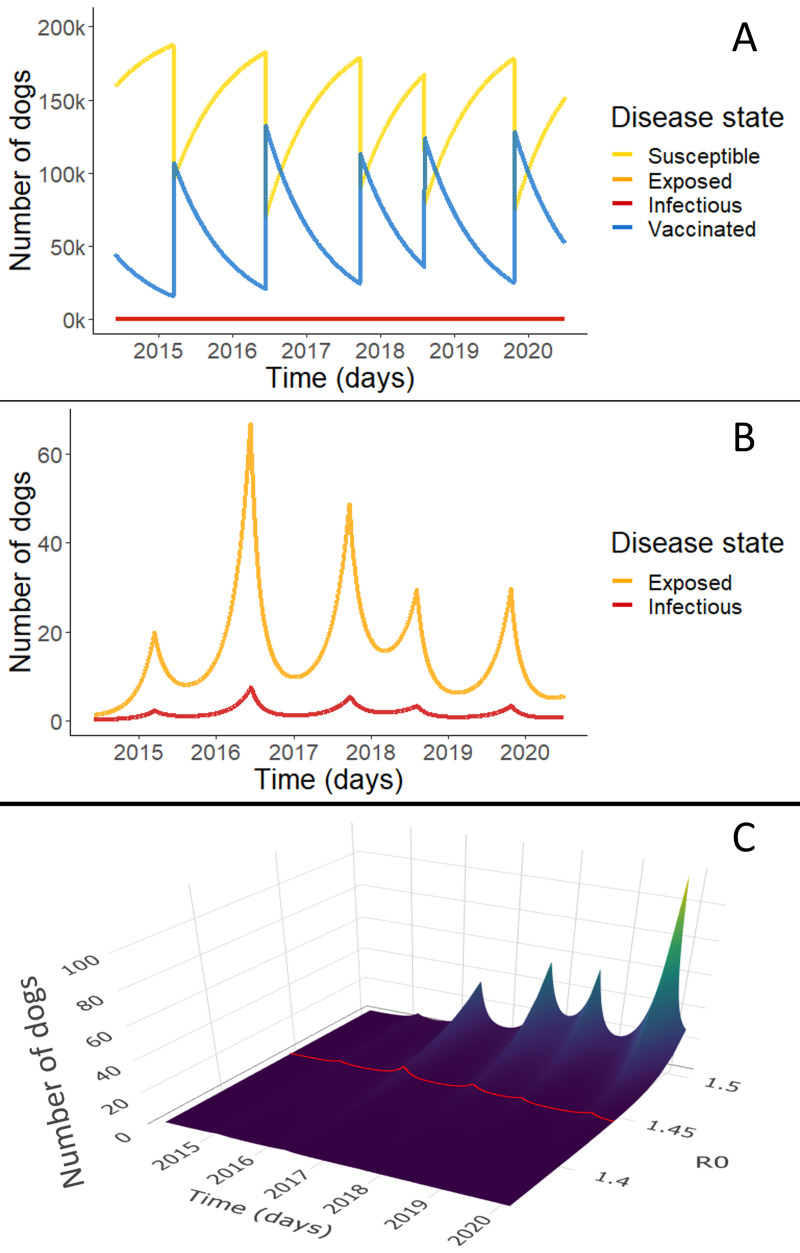
Rabies compartmental model results. Simulations depict daily point prevalence of each disease state (S,E,I,V). Panel A shows the dynamics of all disease states in the best fit rabies model for Arequipa, Peru. The blue line shows the vaccinated dog population numbers over time and the yellow line shows the susceptible population dynamics. Because the proportion of rabies exposed (pink line) and infected dogs (red line) is so small, these dynamics are not apparent in Panel A. Panel B highlights these exposed and infectious dynamics with an adjusted scale. Panel C shows infected population dynamics for a range of R_0_ from 1.36–1.5. In other words, it represents the red line of infected population dynamics shown in B but for a range of R_0_. The trends extend for the full range of possible values of R_0_ [1.36, 2.0] which can be seen in the supplementary information (Figure D in S2 Appendix).

There were 214 reports of rabid dogs in the department of Arequipa from March 2015 to March 2020 out of 2,650 samples submitted over the period (Figure B and Figure C in S2 Appendix), on average a little less than one (0.93) per week. Our model has reasonable matching to the reported case data assuming a reporting rate of 10% and an R_0_ of 1.44 (Figure B in S2 Appendix). The full dynamics (without any scaling for underreporting) can be seen in Fig 1, clearly showing the cyclic nature of immunity and transmission caused by the yearly vaccination campaigns. Population immunity provided by the yearly vaccination campaign decays quickly due to high rates of population turnover (controlled by parameters μ and θ) and loss of vaccine-provided immunity (ν_*2*_) (Fig 1A). The proportion of the population affected by rabies virus transmission is so small that it is not apparent when shown together with the susceptible and vaccinated population (Fig 1A). However, the isolated exposed and infectious population dynamics follow a cyclic pattern (Fig 1B) caused by the pulses of immunity and subsequent decay: waves of exposed and infected dogs rise as population immunity falls. We also examined this behavior over a select range of possible values of R_0_ (Fig 1C) and though the amplitude of peaks may change, the rising trends remain the same. The trends are consistent for the full range of possible values of R_0_ from 1.36–2.0 (Figure D in S2 Appendix).

**Fig 2:**
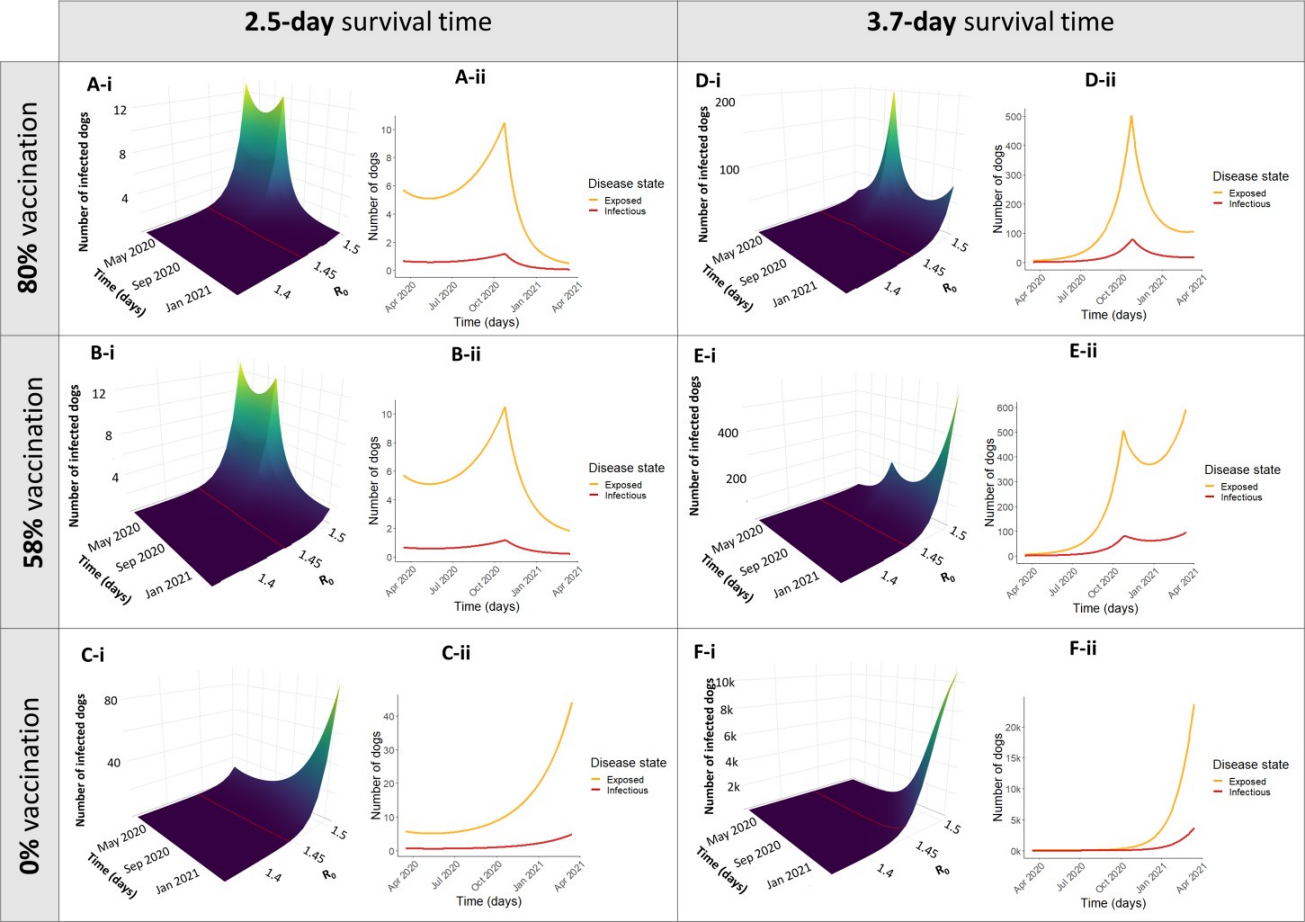
Different simulations of disruption scenarios for 1 year. Simulations depict daily point prevalence of each disease state (S,E,I,V). Simulations were run for 1 year after the beginning of COVID-19 control measures in Arequipa, Peru (March 16, 2020- March 16, 2021). Panels A-C depict different vaccination scenarios with normal levels of surveillance and control measures leading to an average survival time (ST) of rabid dogs to be 2.5 days. Panels D-F show the same vaccination scenarios with decreased surveillance leading to an increased survival time of rabid dogs to 3.7 days. The vaccination scenarios depicted correspond to yearly vaccination campaigns reaching the optimal goal of 80% coverage (Panels A, D), a sub-optimal level of 58% coverage (Panels B, E), and a complete cancellation of the vaccination campaign where coverage is 0% (Panels C, F). Both the surface plots with a range of values of R_0_ (i) and a transect where R_0_ = 1.44 (ii) are displayed).

The effects of the disruption of mass vaccination and surveillance are displayed both as a surface plot with the full range of possible values of R_0_, and as a 2D line plot with R_0_ = 1.44, representing our best estimate for Arequipa, Peru (Fig 2). With zero dogs vaccinated in the city (due to a cancelled vaccination campaign), cases begin to grow exponentially (Fig 2C and 2F). In the ideal case that vaccine coverage reached the 80% recommended by PAHO, the numbers of infected dogs were suppressed to nearly 0 (Fig 2A and 2D). However, even intermediate coverage of 58% has a significant impact on suppressing the rise in infected numbers (Fig 2B and 2E) compared to no vaccination coverage at all (Fig 2C and 2F). The effect of decreased surveillance and subsequent focus control is postulated to result in increased rabid dog survival time from 2.5 to 3.7 days as seen in Fig 2D, 2E and 2F though incidence increases, the number of infected dogs can still be dampened by mass vaccination. The worst-case scenario, where all control activities, mass dog vaccination, surveillance, and focus control, cease, results in a marked exponential rise in rabies cases within a few months (Fig 2E).

**Fig 3:**
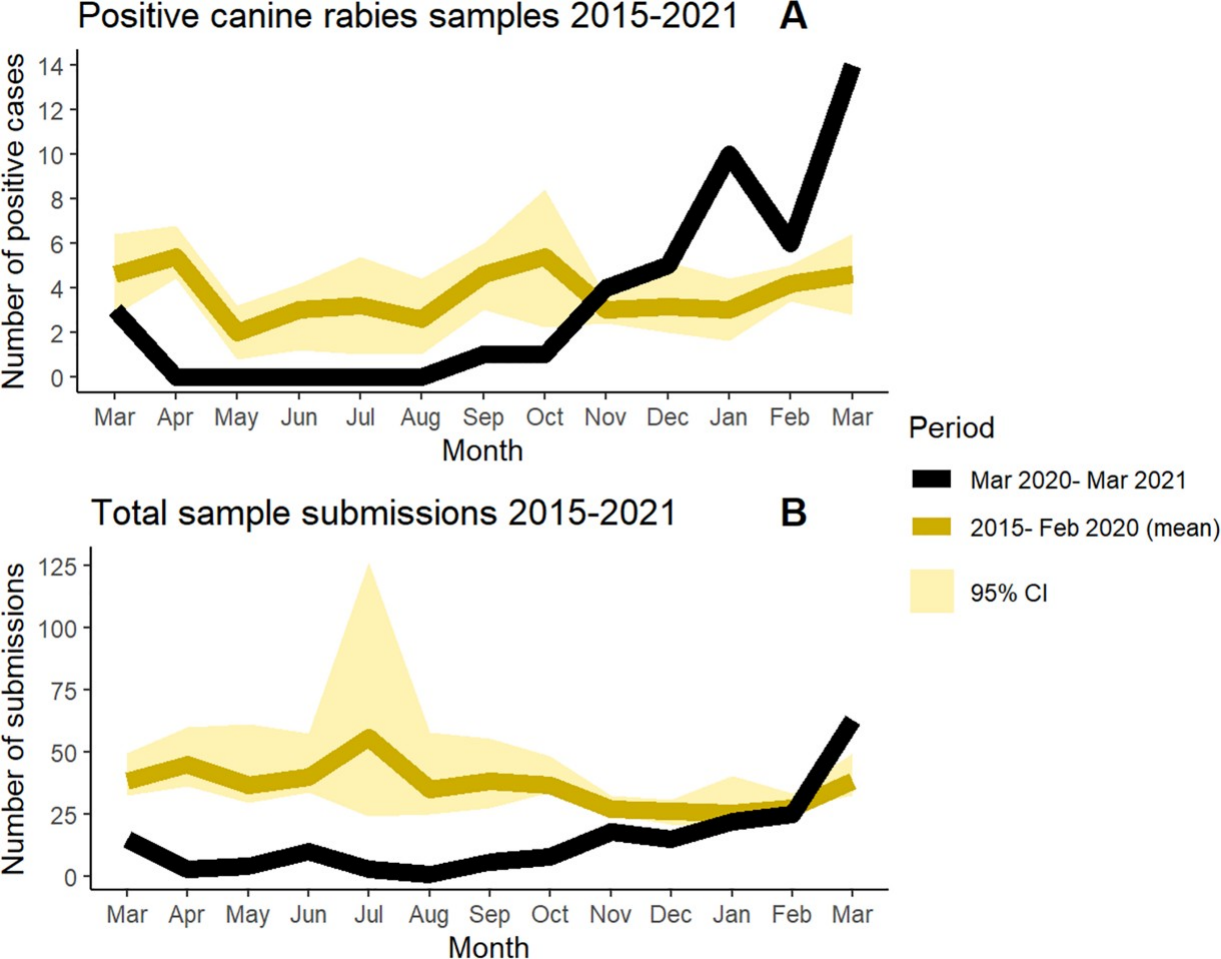
Canine rabies diagnostic samples submitted from 2015–2021. Averages for monthly canine rabies case counts (monthly cumulative incidence) confirmed by laboratory diagnosis (Panel A) and for total canine diagnostic sample submissions to the Peru Ministry of Health for Arequipa Department (Panel B) were calculated by aggregating monthly data from March 2015 (when rabies was first reported in Arequipa city) to February 2020 (the last month that Peru was operating under non-COVID conditions) and compared to pandemic time surveillance data (March 2020 to March 2021). 95% confidence intervals were computed via bootstrap methods by resampling 2000 times. A full timeline is available in the supplementary information (Figure C in S2 Appendix).

**Fig 4:**
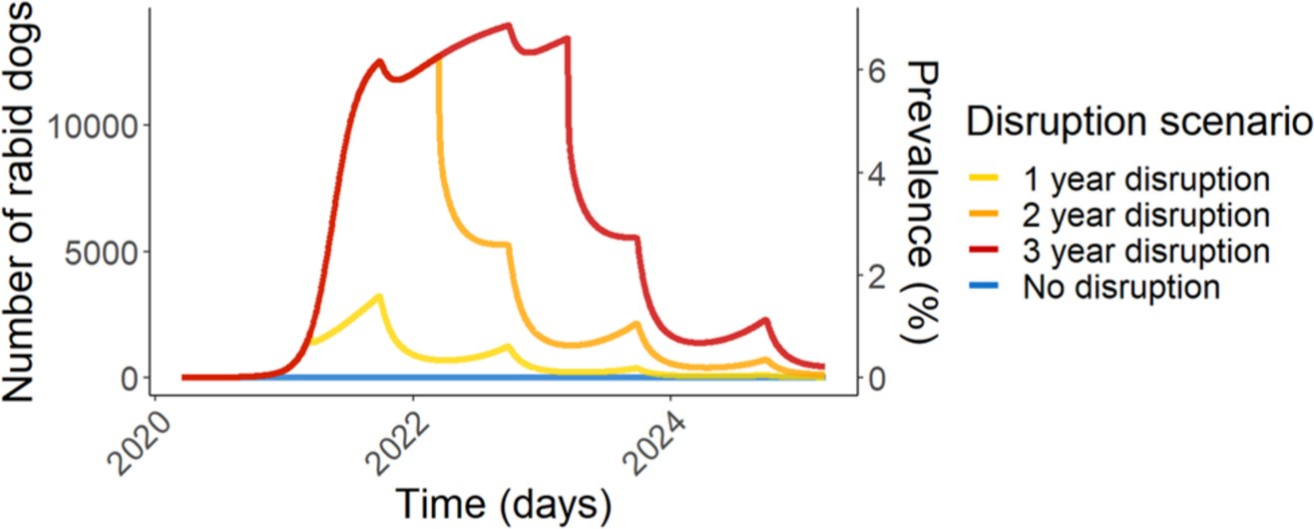
Disruption scenario simulations for 5 years. Rabies dynamics in Arequipa were simulated for five years under four different scenarios. The “No disruption” scenario depicts a counterfactual no-covid scenario where vaccination campaigns were conducted at pre-pandemic normal (58% coverage) and average surveillance remained normal (average survival time of rabid dogs = 2.5 days) throughout the simulated time period (March 2020-March 2025). The disruption scenarios simulate 1–3 years of COVID-19 disruptions with vaccination coverage reduced to 12.3% and average survival time of rabid dogs increased to (3.7 days) before returning to pre-pandemic normal.

**Fig 5:**
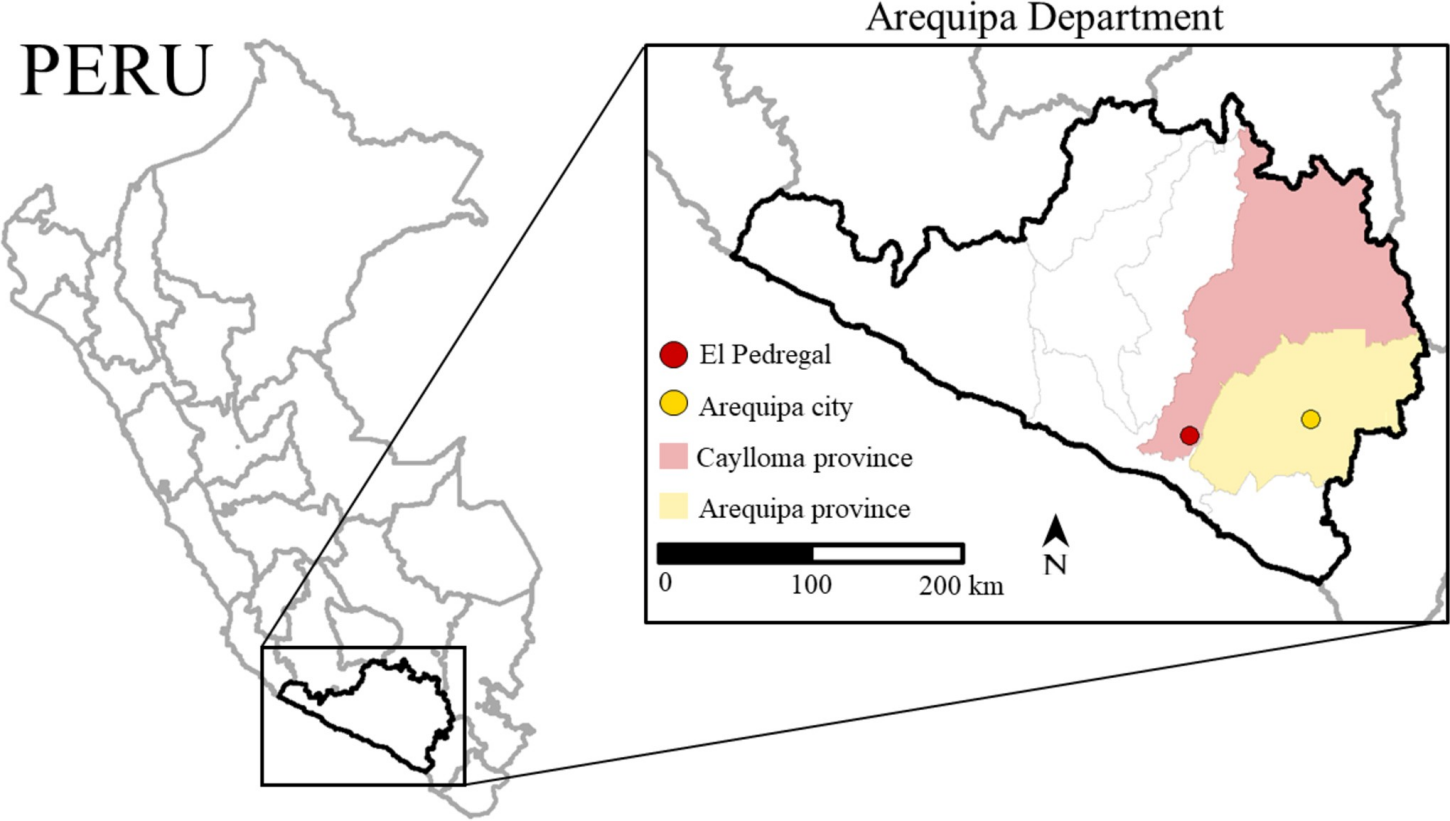
Map of Arequipa Department. Arequipa Department consists of 8 provinces. Prior to February 2021, rabies cases were contained in Arequipa province (the province containing Arequipa city). In February 2021, cases spread to the neighboring province of Caylloma, primarily in the city of El Pedregal. Seven cases have since been detected in El Pedregal. Shapefiles used to create maps are from Peru’s National Geo-referenced Data Platform Geo Peru [32].

In Arequipa, the 2020 vaccination campaign was severely reduced, reaching a city-wide coverage of only 12.3%. Surveillance, measured by number of samples submitted to the ministry of health was also severely reduced in 2020 compared to previous years averages (Fig 3). Under these conditions model trends predict an exponential rise in rabies cases within months, a prediction that has been unfortunately corroborated through preliminary surveillance results from December 2020 through March 2021. From November 2020 to February 2021 higher than average numbers of cases were detected despite continued reduced surveillance effort and only a small number of sample submissions compared to previous years (Fig 3). March 2021 had the largest number of canine rabies cases detected (14 cases) since the virus was re-introduced in 2015. Intermediate-term effects (5 years) were simulated in Fig 4, showing continued dramatically increased simulated rabies burden due to COVID-19 disruptions. Moreover, the rabies affected area has extended. From 2015 to January 2021, dog rabies cases were contained within the city of Arequipa, province of Arequipa (Fig 5). Since February 2021, rabies has expanded within the department of Arequipa to a neighboring province; at least 7 rabid dogs have been detected in the province of Caylloma (Fig 5).

## Discussion

Our model results predicted that disruptions to rabies control programs caused by the COVID-19 pandemic would substantially increase the number of cases of canine rabies with an associated increased risk of human rabies within a few months. Preliminary surveillance trends from Arequipa, Peru suggest that an increased rise in canine rabies cases is already occurring. This crisis has the potential to reverse strides towards the regional goal of zero canine-mediated human deaths of rabies by 2030 [33]. Given that COVID-19 will continue to challenge public health departments in the short- and medium-term as public health officials work to immunize populations worldwide, it is essential to create a strategy for rabies surveillance and prevention during the COVID-19 pandemic. This strategy should consider new approaches to dog vaccination that can accommodate social distancing and other COVID-19 prevention measures. New dog vaccination approaches, even with suboptimal coverage, could reduce canine rabies cases in the short term and prevent dog-mediated human rabies.

Our analysis and the sharp rise in canine rabies cases in Peru have broad implications regionally for Latin America. Our model of canine rabies in Arequipa demonstrates the effects of COVID-19 on the spread of canine rabies in a city with a medium to large human population (about 1 million), active immigration and emigration, continuous but suboptimal efforts to control rabies, and a fairly large free-roaming dog population [22,31,34]; many of these characteristics are shared with other Latin American urban areas. Also, Arequipa represents an area of rabies reintroduction and rabies re-establishment [34], both undesired and rare events. Modeling a city with these characteristics and continuous rabies virus transmission may provide insight into rabies-endemic Latin American cities during the COVID-19 pandemic and also into the risk of expansion to non-endemic neighboring cities.

In rabies-affected areas in Latin America, the COVID-19 pandemic has disrupted the multi-pronged rabies control program at several points, the full effects of which may not become apparent for several years. The first prong of rabies control programs that may be affected by COVID-19 is the rabies surveillance system. Because rabies surveillance systems rely heavily on submission of samples from dogs reported to exhibit clinical signs of rabies by the public, the absence of people leaving home to observe these dogs has caused greatly decreased reporting rates. In Arequipa, from April 2020 to September 2020, an average of 4.7 samples were submitted per month, compared to an average of 35.7 previously. We postulate that dogs may live longer and transmit rabies virus to a larger number of dogs before they die, as reflected by a decreased parameter α, the death rate due to rabies.

The second prong of rabies control in Arequipa is yearly vaccination campaigns. Due to COVID-19, the yearly dog vaccination campaign in Arequipa in 2020 was severely reduced to a coverage rate of 12.3%, and similar disruptions occurred across Latin America [17–21]. COVID-19 poses challenges to rabies vaccination campaigns in a few ways. First, geographic areas with high rates of canine rabies in particular need of vaccination points also tend to be areas with high rates of COVID-19 due to population density, which leads to concern for the safety of healthcare personnel and dog owners. Second, public health organizations are focusing their energy and resources on the COVID-19 crisis at hand. Diverting scarce public health resources towards a crisis is often necessary for some amount of time; however, our model suggests that reducing or postponing vaccination campaigns could have detrimental consequences on the spread of dog rabies and, ultimately, public health. The 2020 vaccination campaign in Arequipa covered only 4 districts out of 14 in the city; patchy, heterogeneous coverage likely further exasperates case surges seen from low vaccination rates [7,22,35–37]. Our results correspond with other studies around the world of the detrimental effects of disrupting rabies mass vaccination campaigns. A modeling study in Chad found that vaccination campaigns needed to be repeated due to imported cases [38], another modeling study in Bali, Indonesia, simulated a rise in cases after control activities were halted [36], and such a rise in rabies cases were observed in South Africa after vaccination campaigns were halted [39]. Even if the recommended 80% vaccination coverage goals may be unattainable, our model indicates that an intermediate (suboptimal) effort can still have a tremendous effect in curbing the rise of canine rabies.

The model presented above has many sources of uncertainty, perhaps the most significant is a likely massive underreporting of canine rabies cases leading to lack of accurate data and bias in parameterized data around which the model is constructed. Inadequate surveillance can exacerbate underreporting—a common problem in rabies- affected areas [3,8,34,40–42]—and lead to more cases as the virus spreads undetected. Additionally, we made several assumptions about the model parameters, that: 1) in Arequipa, 10% of canine rabies cases are detected, 2) the majority of dog bites transmitting rabies virus occur after the onset of rabies clinical signs in the biting (rabid) dog, and 3) vaccination immunity decay follows the conservative licensure estimate though the true decay rate of immunity may be slower. The COVID-19 pandemic likely has caused many changes in both human and dog behavior not captured in our model, such as varied movement and behavior patterns of humans leading to varied scavenging patterns and contact networks by dogs. Finally, our models are not intended for use in predicting exact values on specific dates but rather as tools to assess trends resulting from different control strategies.

The effects of stopping or pausing rabies prevention activities have had serious effects on cases of canine rabies, and consequently, on the risk of human rabies. This problem is not unique to rabies in Latin America; worldwide, COVID-19 is disrupting control efforts in a plethora of infectious disease programs. Already reported in the literature are flagging efforts to control measles and Hepatitis B Virus, decreased surveillance jeopardizing poliovirus eradication goals, and predicted surges in malaria and Dengue fever cases following pandemic neglect [29,43–47]. Animal disease control programs including African Swine Fever and tuberculosis surveillance systems are also at risk [48]. Parallels can be drawn to other public health crises such as Ebola, where protocols to contain Ebola interrupted screening and diagnosis of malaria, tuberculosis, and HIV [49,50]. Adequate surveillance, especially animal disease surveillance, is one essential piece of infectious disease control programs that is often neglected for more immediate emergencies [51–53]. COVID-19 has had a devastating toll on health through serious to fatal cases and economic hardships. However, the true extent of the damage caused by the pandemic may not be observed for years to decades as previously under-control diseases surge due to neglected control programs.

## Supporting information

S1 Alternative Language Abstract Translation of the Abstract in Spanish by Ricardo Castillo-Neyra. (DOCX)

S2 Appendix (DOCX)
