# Supplementary material for: The impact of the COVID-19 pandemic on rabies reemergence in Latin America: The case of Arequipa, Peru
Source: PLoS Negl Trop Dis. 2021 May 21;15(5):e0009414. doi: 10.1371/journal.pntd.0009414 (PMC8174740; doi:10.1371/journal.pntd.0009414)
Supplement: S1 Alternative Language Abstract — (DOCX) [file pntd.0009414.s001.docx]

# **S1 Alternative Language Abstrac**t –

# Translation of the Abstract into Spanish by Ricardo Castillo-Neyra

# **Resumen**

**Antecedentes:** En Latinoamérica ha habido un progreso tremendo hacia la eliminación de la rabia canina. Los principales componentes de los programas de eliminación de la rabia han sido la vigilancia de perros rabiosos y campañas anuales ininterrumpidas de vacunación canina masiva. Desafortunadamente, las medidas esenciales para controlar la COVID-19 han tenido el balance negativo de poner en peligro estas actividades de prevención y eliminación de rabia. Nuestro objetivo fue evaluar el efecto que tendría la disrupción de la vigilancia de rabia canina y de la vacunación masiva antirrábica canina sobre las tendencias de casos de rabia.

**Métodos:** Modelamos la dinámica de la rabia canina mediante un modelo determinístico de compartimientos para crear el marco conceptual sobre el cual evaluar como las diferentes disrupciones afectarían la transmisión del virus de la rabia. El modelo fue parametrizado para las condiciones que se encuentran en Arequipa, Perú, una ciudad con transmisión activa de virus rábico. Examinamos nuestros resultados sobre un rango de valores plausibles de R_0_ (1.36-2.0). Además, evaluamos prospectivamente datos de vigilancia durante la pandemia para detectar cambios temporales.

**Resultados:** Nuestro modelo sugiere que una disminución en la cobertura de vacunación canina, así como una disminución en la vigilancia, llevarían a un aumento súbito de rabia canina en solo meses. Estos resultados son consistentes sobre todos los valores plausibles de R_0_. Nuestros datos de vigilancia de finales del 2020 y comienzos del 2021 de Arequipa, Peru, confirman que los casos de rabia canina van en rápido aumento.

**Conclusión:** Estas tendencias en Arequipa, si algo indican sobre la región, sugieren que los logros alcanzados en Latinoamérica hacia la eliminación de la rabia humana mediada por perros se encuentran en peligro.
