# Supplementary material for: The impact of the COVID-19 pandemic on rabies reemergence in Latin America: The case of Arequipa, Peru
Source: PLoS Negl Trop Dis. 2021 May 21;15(5):e0009414. doi: 10.1371/journal.pntd.0009414 (PMC8174740; doi:10.1371/journal.pntd.0009414)
Supplement: S2 Appendix — (DOCX) [file pntd.0009414.s002.docx]

# **S2 Appendix**

**
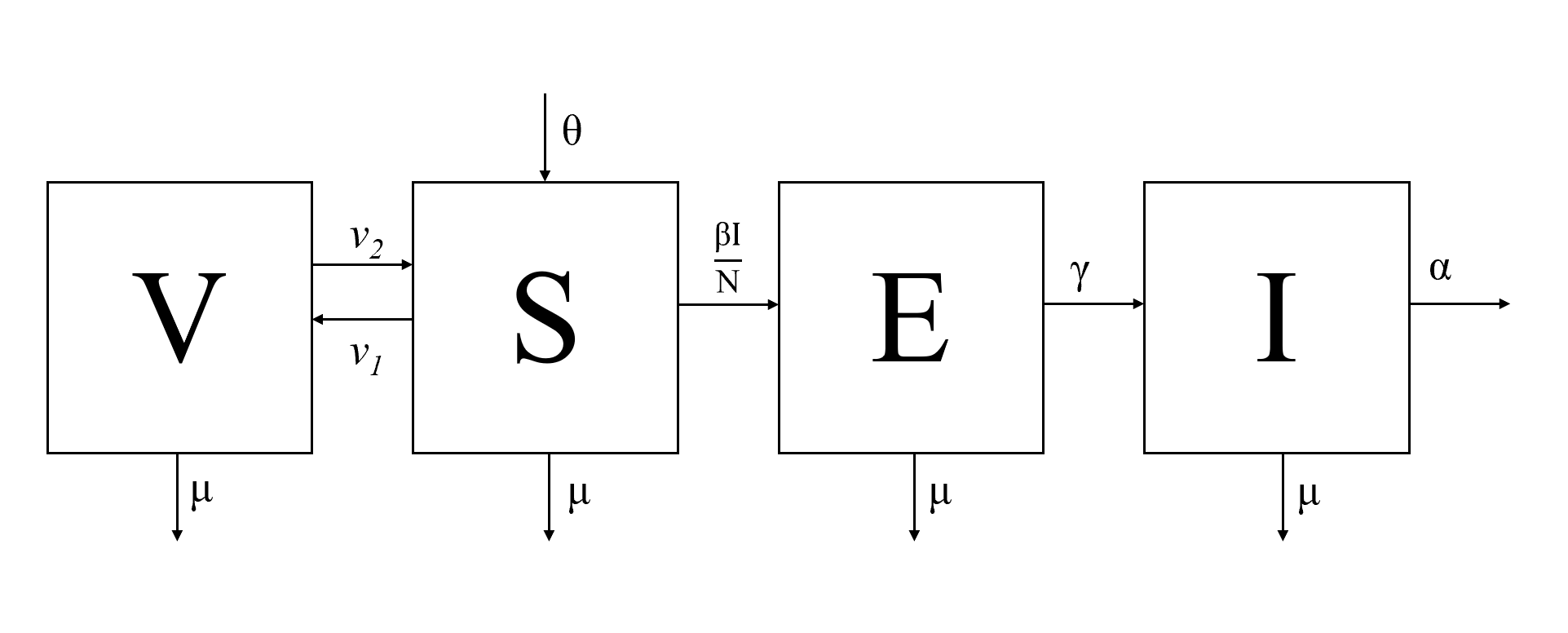
**

**Figure A**

A flow diagram depicting 4 different population states (V-vaccinated, S- susceptible, E-exposed, I-infected) of dogs with arrows depicting the movement of individuals between states.

**Text A**

***Parameter estimations***

The total population, N, was calculated based on estimates provided by the Peruvian Ministry of Health to be 203,183 dogs [(1)](https://www.zotero.org/google-docs/?fnCtYZ). The background death rate, 𝜇, was calculated as the inverse of the average age of the dog population. Average age was calculated from a 2019 survey of over 3000 dogs to be 1099.2 days [1063.3, 1135.1]. Survey methods are described in detail elsewhere [(2)](https://www.zotero.org/google-docs/?Tkkai4). The birth rate, 𝛳, was calculated to maintain a steady state equilibrium: 𝛳 = 𝜇N + 𝛼I.

The incubation period of rabies (1/𝛾) was assumed to be similar to rates reported in the literature. Hampson et al. found the maximum likelihood estimate of the mean incubation period from when exposed dogs were bitten to when they become infectious to be 22.3 days [20.0, 25.0] [(3)](https://www.zotero.org/google-docs/?J397nz). The infectious period (1/𝛼) when surveillance is in place, was estimated from Arequipa focus control data to be 2.5 days [1.9, 3.1] based on the difference between when owners reported their dog began showing symptoms and when they were euthanized by the public health veterinarians or health inspectors.

The instantaneous rate of immunity loss (*v_2_*) is estimated to be the inverse of the immunity period offered by the vaccine. The vaccine used in the vaccination campaigns is rated for 1 year, so correspondingly *v_2_* = 1/365. Vaccination rates vary by year depending on coverage rates of the vaccination campaign. We make the simplifications that immunity is immediate upon vaccination and that the entire vaccination campaign happens in one day (though in reality it is spread across several days to weeks). In this way, immunity is “pulsed” once yearly and then immediately begins to decay. Supplement Table 1 shows estimated coverage rates from longitudinal survey data. Because vaccination coverage rates are based on a few days or a single day (the vaccination campaign) , we assume a single day for our calculations [(4)](https://www.zotero.org/google-docs/?QswTUD).The pulsed vaccination rate (*v_1_*) can be calculated then as vaccination coverage = 1-e^-v1*t^, where t =1 day.

The transmission coefficient, 𝛽, can be very difficult to measure, but can be derived from the equation for R_0_. Using the next generation matrix methods [(5)](https://www.zotero.org/google-docs/?rQiUNt), an equation for R_0_ can be derived from the disease system:

R_0_= 𝛽𝛾/((𝛼+𝜇)(𝛾+𝜇))

One challenge in estimating R_0_ for canine rabies globally is lack of accurate case counts. Rabies cases in humans are grossly underreported; in dogs, this trend is even more apparent [(6–12)](https://www.zotero.org/google-docs/?cYMDyp). The WHO estimated that though in 2010 less than 10 human cases were reported in Latin America, there were in reality closer to 200 (World Health Organization, 2013). In the literature the R_0_ for canine rabies across the world is reported from 1-2 [(3,13–15)](https://www.zotero.org/google-docs/?pFpaVM).

In Arequipa, R_0_ was estimated from data collected from focus control team members responding to positive cases. Out of 214 cases, there are data on 33 cases about the number of secondary dogs that a rabid dog bit. Hampson et al. showed there is about a 0.49 probability that a dog will contract rabies if bitten by a rabid dog with a binomial confidence interval of [0.45, 0.52] (Hampson et al., 2009). To estimate R_0_ for Arequipa, we randomly assigned a probability form the binomial distribution described by Hampson and used a bootstrap resampling method to estimate mean and 95% confidence intervals (Hampson et al., 2009). We estimate R_0_ to be 1.36 [1.05, 1.88]. This R_0_ estimation should be considered as the low end of possible values as the data are biased due to being from focus control reports; these were cases that were responded to and controlled, limiting their number of secondary cases. Furthermore, we calculated R_0_ from 𝛽 by fitting our simulated monthly incidence of infected dogs to the reported rabies case data using a least squares fitting approach [(16,17)](https://www.zotero.org/google-docs/?7K8jIQ). We use a conservatively low estimate of reporting rate (10%) of rabid dog cases. Due to the high number of people killing rabid dogs without reporting them and the high number of dogs hit by cars and never investigated, 10% is likely an overestimation of reporting rate for canine rabies cases in Arequipa. Using this rate of underreporting, we estimated an R_0_ of 1.44.

***References***

[1. Red Arequipa Caylloma. Plan de accion de la campaña de vacunacion antirabica canina 2019 [Internet]. 2019. Available from: https://redperifericaaqp.gob.pe/wp-content/uploads/2019/04/accion-de-la-campana-atirabica-2019-comprimido.pdf](https://www.zotero.org/google-docs/?8rK3LU)

[2. Castillo-Neyra R, Toledo AM, Arevalo-Nieto C, MacDonald H, Puente-León MD la, Naquira-Velarde C, et al. Socio-spatial heterogeneity in participation in mass dog rabies vaccination campaigns, Arequipa, Peru. PLoS Negl Trop Dis. 2019 Aug 1;13(8):e0007600.](https://www.zotero.org/google-docs/?8rK3LU)

[3. Hampson K, Dushoff J, Cleaveland S, Haydon DT, Kaare M, Packer C, et al. Transmission Dynamics and Prospects for the Elimination of Canine Rabies. PLOS Biol. 2009 Mar 10;7(3):e1000053.](https://www.zotero.org/google-docs/?8rK3LU)

[4. Castillo-Neyra R, Brown J, Borrini K, Arevalo C, Levy MZ, Buttenheim A, et al. Barriers to dog rabies vaccination during an urban rabies outbreak: Qualitative findings from Arequipa, Peru. PLoS Negl Trop Dis. 2017 Mar 17;11(3):e0005460.](https://www.zotero.org/google-docs/?8rK3LU)

[5. Diekmann O, Heesterbeek JAP, Roberts MG. The construction of next-generation matrices for compartmental epidemic models. J R Soc Interface. 2010 Jun 6;7(47):873–85.](https://www.zotero.org/google-docs/?8rK3LU)

[6. Banyard AC, Horton DL, Freuling C, Müller T, Fooks AR. Control and prevention of canine rabies: the need for building laboratory-based surveillance capacity. Antiviral Res. 2013 Jun;98(3):357–64.](https://www.zotero.org/google-docs/?8rK3LU)

[7. Benavides JA, Megid J, Campos A, Rocha S, Vigilato MAN, Hampson K. An evaluation of Brazil’s surveillance and prophylaxis of canine rabies between 2008 and 2017. PLoS Negl Trop Dis. 2019 Aug 5;13(8):e0007564.](https://www.zotero.org/google-docs/?8rK3LU)

[8. Carvalho MF de, Vigilato MAN, Pompei JA, Rocha F, Vokaty A, Molina-Flores B, et al. Rabies in the Americas: 1998-2014. PLoS Negl Trop Dis. 2018 Mar 20;12(3):e0006271.](https://www.zotero.org/google-docs/?8rK3LU)

[9. Castillo-Neyra R, Zegarra E, Monroy Y, Bernedo RF, Cornejo-Rosello I, Paz-Soldan VA, et al. Spatial Association of Canine Rabies Outbreak and Ecological Urban Corridors, Arequipa, Peru. Trop Med Infect Dis [Internet]. 2017 Aug 13 [cited 2020 Jul 11];2(3). Available from: https://www.ncbi.nlm.nih.gov/pmc/articles/PMC6082090/](https://www.zotero.org/google-docs/?8rK3LU)

[10. Nel LH. Discrepancies in Data Reporting for Rabies, Africa. Emerg Infect Dis. 2013 Apr;19(4):529–33.](https://www.zotero.org/google-docs/?8rK3LU)

[11. Vigilato MAN, Clavijo A, Knobl T, Silva HMT, Cosivi O, Schneider MC, et al. Progress towards eliminating canine rabies: policies and perspectives from Latin America and the Caribbean. Philos Trans R Soc B Biol Sci [Internet]. 2013 Aug 5 [cited 2020 Jun 18];368(1623). Available from: https://www.ncbi.nlm.nih.gov/pmc/articles/PMC3720041/](https://www.zotero.org/google-docs/?8rK3LU)

[12. Widdowson M-A, Morales GJ, Chaves S, McGrane J. Epidemiology of Urban Canine Rabies, Santa Cruz, Bolivia, 1972–1997. Emerg Infect Dis. 2002 May;8(5):458–61.](https://www.zotero.org/google-docs/?8rK3LU)

[13. Coleman PG, Dye C. Immunization coverage required to prevent outbreaks of dog rabies. Vaccine. 1996 Feb 1;14(3):185–6.](https://www.zotero.org/google-docs/?8rK3LU)

[14. Kitala PM, McDermott JJ, Coleman PG, Dye C. Comparison of vaccination strategies for the control of dog rabies in Machakos District, Kenya. Epidemiol Infect. 2002 Aug;129(1):215–22.](https://www.zotero.org/google-docs/?8rK3LU)

[15. Knobel DL, Lembo T, Morters M, Townsend SE, Cleaveland S, Hampson K. Chapter 17 - Dog Rabies and Its Control. In: Jackson AC, editor. Rabies (Third Edition) [Internet]. Boston: Academic Press; 2013 [cited 2020 Jul 2]. p. 591–615. Available from: http://www.sciencedirect.com/science/article/pii/B9780123965479000171](https://www.zotero.org/google-docs/?8rK3LU)

[16. Huang J, Ruan S, Shu Y, Wu X. Modeling the Transmission Dynamics of Rabies for Dog, Chinese Ferret Badger and Human Interactions in Zhejiang Province, China. Bull Math Biol. 2019 Apr 1;81(4):939–62.](https://www.zotero.org/google-docs/?8rK3LU)

[17. Zhang J, Jin Z, Sun G-Q, Zhou T, Ruan S. Analysis of Rabies in China: Transmission Dynamics and Control. PLOS ONE. 2011 Jul 14;6(7):e20891.](https://www.zotero.org/google-docs/?8rK3LU)

**
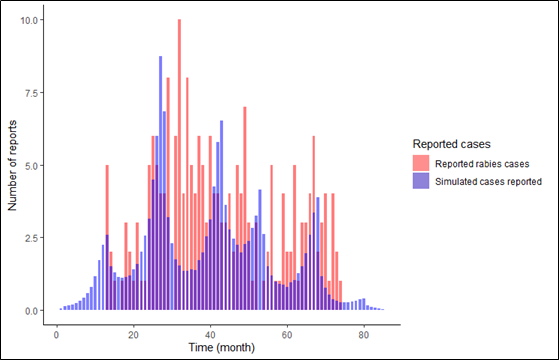
**

**Figure B**

Reported rabies case data are depicted in pink bars and rabies cases simulated by the model are depicted in blue. The model is parameterized by fitting monthly incidence assuming a 10% reporting rate.

**Table A**

Mass dog vaccination campaign coverage (2015-2019).

| **Year** | **Coverage estimate** |
| --- | --- |
| 2014 | 48.98% |
| 2015 | *48.98% |
| 2016 | 61.48% |
| 2017 | 49.90% |
| 2018 | 52.85% |
| 2019 | 58.5% |

*Due to lack of reliable data, we relied on the estimate from 2014 for 2015

**
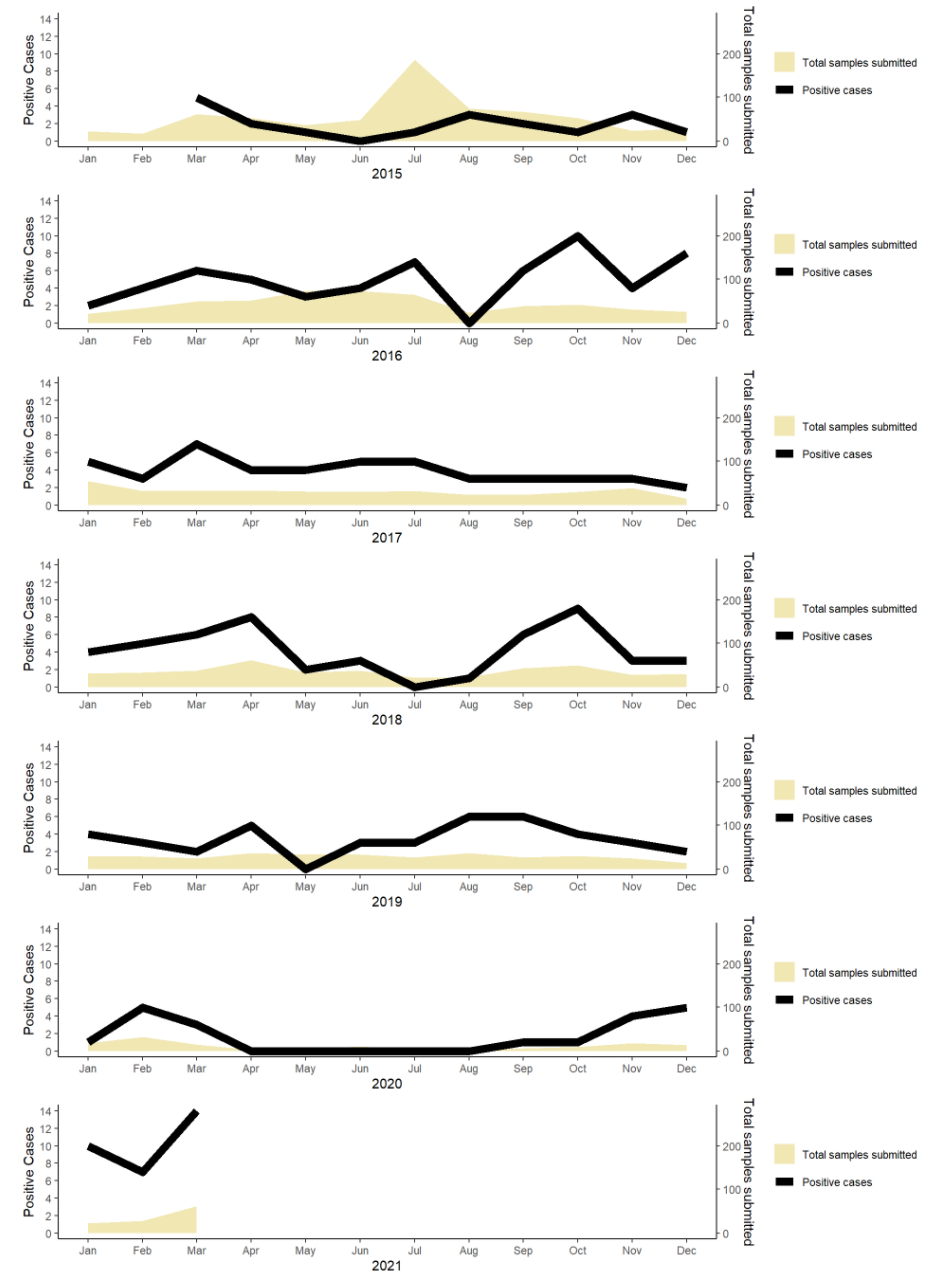
**

**Figure C**

Timeline of surveillance and cases of canine rabies from 2015 to March 31, 2021. Plotted are cumulative incidence per month (black line- left axis) and the total number of samples submitted per month (yellow shading- right axis).


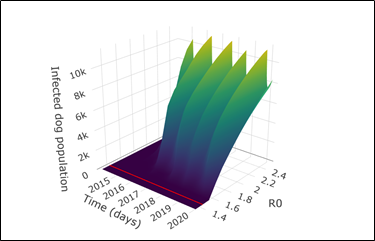


**Figure D**

The simulated number of infected dogs for a wide range of R0. We show all possible values from 1.36, the low estimate found from focus control data to 2.0, a high-end estimate from the rabies literature. The red line shows the transect R0=1.44 which is our best fit estimate for Arequipa. Though the scale makes the line look flat in this image, in Figure 1C in the main text the regional dynamics are displayed at a narrower range.
